# Effects of environmental features and sport hunting on caribou migration in northwestern Alaska

**DOI:** 10.1186/s40462-017-0095-z

**Published:** 2017-03-01

**Authors:** Timothy J. Fullman, Kyle Joly, Andrew Ackerman

**Affiliations:** 1 The Wilderness Society, 705 Christensen Drive, Anchorage, AK 99501 USA; 2 National Park Service, Gates of the Arctic National Park and Preserve, Arctic Inventory and Monitoring Network, 4175 Geist Rd., Fairbanks, AK 99709 USA; 3 National Park Service, Western Arctic National Parklands, 4175 Geist Rd., Fairbanks, AK 99709 USA

**Keywords:** Aircraft, Alaska, Caribou, Hunting, Migration, Movement, Noatak National Preserve, *Rangifer tarandus*, Resource selection

## Abstract

**Background:**

Ungulate movements are influenced by a variety of biotic and abiotic factors, which may affect connectivity between key resource areas and seasonal ranges. In northwestern Alaska, one important question regarding human impacts on ungulate movement involves caribou (*Rangifer tarandus*) response to autumn hunting and related aircraft activity. While concerns have been voiced by local hunters about the influence of transporter aircraft and non-local sport hunters, there has been little quantitative analysis of the effects of hunter activity on caribou movement. We utilized a novel spatial dataset of commercial aircraft landing locations and sport hunter camps in and around Noatak National Preserve to analyze resource selection of caribou in autumn for non-local hunting activity and environmental features. We combined step selection functions with randomized shortest paths to investigate whether terrain ruggedness, river width, land cover, and hunting activity (in the form of aircraft landings and sport hunter camps) facilitated or impeded caribou movement. By varying a parameter in the randomized shortest path models, we also explored the tradeoff between exploration and exploitation in movement behavior exhibited by traveling caribou.

**Results:**

We found that caribou avoided rugged terrain and areas with more river, forest, and tall shrubs while selecting for areas dominated by tussock tundra and dwarf shrubs. Migration of caribou through Noatak does not appear to be inhibited by sport hunting activity, though this does not preclude the possibility of temporary effects altering availability of caribou for individual hunters. Caribou exhibited exploratory movement, following predictions of a random walk model. This behavior may facilitate the location of remaining patches of high-quality forage prior to the onset of winter, especially during mild autumns.

**Conclusions:**

Understanding animal movement behavior is fundamental to protecting critical areas of connectivity and to informing management decisions. Our study identifies migratory connectivity and hotspots of potential conflict among user groups, enabling development of policies that balance human access with species conservation.

**Electronic supplementary material:**

The online version of this article (doi:10.1186/s40462-017-0095-z) contains supplementary material, which is available to authorized users.

## Background

Animal movement is a key ecological process [1–3] that influences population dynamics [4, 5], predator-prey interactions [6, 7], gene flow [8, 9], use of dynamic resources [10], and vulnerability to environmental change [11–13]. In light of extensive and increasing human alteration of natural areas, maintaining connectivity across landscapes is important to facilitate species movement and retain the associated processes [14–16]. A strong understanding of animal movement behavior plays a critical role in assessing connectivity, as well as how environmental changes might enhance or reduce connectivity [17].

Studies of animal movement and connectivity are increasingly being used to inform management decisions [18, 19]. Resource managers are often tasked with conserving species and maintaining habitat while balancing the interests of multiple user groups. This is an inherently spatiotemporal process that involves choices about where, when, and how access should be given versus restricted to meet management objectives. The importance of protected areas in helping maintain connectivity for wildlife [20–22] makes it critical that such decisions do not unduly compromise the ability of species to move through managed areas.

In northern Alaska, caribou (*Rangifer tarandus*) form an iconic part of the landscape, migrating thousands of kilometers each year in one of the longest terrestrial migrations in North America [23, 24]. Caribou migrate north in the spring to calve in the northern foothills of the Brooks Range [25, 26]. In autumn, most caribou migrate south to winter in areas with greater cover and winter food availability [27, 28]. As the most abundant large herbivores in the Arctic [29], caribou are an important food resource for a variety of predator species [30–33] as well as local communities [34–36]. Non-local sport hunters also harvest caribou in the region [37]. Balancing the subsistence needs of local hunters with the responsibility to provide hunting opportunities for non-local hunters can be a difficult task for managers. In some areas, local hunters have come into conflict with non-local hunters seeking caribou [38, 39]. Though the documented impacts typically have been relatively minor and of short duration (e.g., [40, 41]), local hunters perceive that non-local hunting and the associated aircraft activity have changed the migratory patterns of caribou [42]. Agencies tasked with managing wildlife species and human access to protected areas need additional information regarding critical movement routes of subsistence species like caribou, as well as an increased understanding of animal response to human activity to identify “hotspots” of potential conflict among user groups.

We investigate movement patterns of caribou in response to non-local sport hunting and environmental features in Noatak National Preserve, Alaska (hereafter, Noatak). Caribou have been hunted in Noatak for thousands of years by local indigenous people. Sport hunting in and around Noatak has occurred for decades but appears to have increased markedly since 2000 [37]. Local hunters primarily access Noatak using boats along the Noatak River, while sport hunters typically use small, commercially-operated transporter aircraft [37]. Conflict between the two groups arose early in Noatak’s history over competition for caribou and other species and the perceived negative effects of aircraft noise by local hunters [39]. Similar concerns continue to be voiced today [42, 43]. In response to concerns about user conflict, federal land managers established a delayed entry area for non-local hunters in the western portion of Noatak to provide increased opportunities for undisturbed hunting by local subsistence hunters. In 2016, faced with continued reports of user conflict and declining population size of the caribou herd using Noatak, the Federal Subsistence Board closed Noatak and other federal public lands to sport hunters seeking to harvest caribou.

The purpose of this study is to investigate how a suite of factors, especially sport hunting activity, affect caribou movement patterns through Noatak. An increased understanding of whether the ability of caribou to pass through Noatak during their autumn migration is altered by sport hunting activity can be used to help inform decisions by land management agencies, such as continuation of public lands closures for certain user groups like caribou sport hunters. We use caribou GPS telemetry data and datasets of sport hunter camps and aircraft landing sites over a period of four hunting seasons to evaluate the hypothesis that sport hunting activity influences habitat selection of caribou migrating through Noatak against the null hypothesis of no impact.

In addition to exploring the influence of sport hunting activity on caribou movement, we test how observed caribou movement patterns correspond to a range of possible movement behaviors. Common techniques for assessing movement routes and connectivity of wildlife species, such as least cost path [44] and circuit theory [45] models, have contributed to understanding of connectivity [46–49], but have at times been criticized for making unrealistic movement assumptions [50–53]. Further empirical evidence is needed of how animal movement behavior coincides with different movement strategies to inform model choice for connectivity studies [51, 54]. We use the step selection function randomized shortest path (SSF-RSP) approach [53] to identify whether caribou movement behavior most closely fits a random walk movement pattern (in line with circuit theory), an optimal movement strategy (in line with least cost paths), or an intermediate strategy that mixes the two. The results of these analyses can help inform future connectivity modeling work as well as providing specific information to Alaskan managers seeking to balance hunter access from different user groups with species conservation.

## Methods

### Study area

**Fig. 1:**
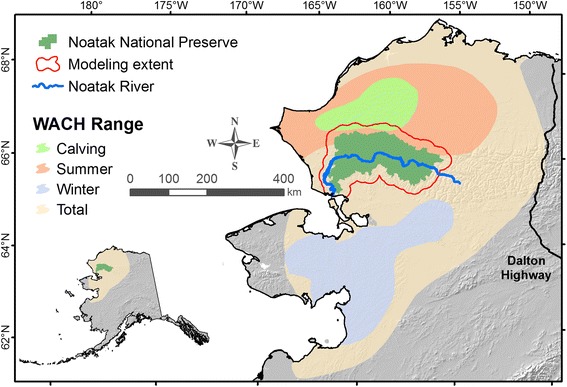
Noatak National Preserve study area in northwestern Alaska. Caribou from the Western Arctic Caribou Herd move through the preserve in autumn and cross the Noatak River, which is heavily used by both local and non-local hunters. Analyses focused on the area within Noatak, but model runs included a 20 km buffer around the preserve to avoid edge effects in resistance modeling. Range map courtesy of the Alaska Department of Fish and Game

Noatak spans approximately 2.7 million hectares in northwestern Alaska (Fig. 1). Along with neighboring Gates of the Arctic National Park and Preserve, Noatak protects the watershed of the Noatak River, the longest free-flowing wild river in the United States [55]. Noatak is used by caribou of the Western Arctic Caribou Herd (WACH) throughout the year, and especially during their spring and autumn migrations when they pass through Noatak on their way to the calving grounds and winter range, respectively [24, 28]. Caribou are a key species of management concern in Noatak [55], with a focus on maintaining both healthy ecological conditions for caribou and a sustainable population for local subsistence hunters. The WACH currently numbers around 201,000 individuals [56] and has been in decline since the early 2000s [28]. Approximately 12,000 caribou are harvested from the WACH each year, with about 5% of this harvest occurring by non-local hunters [57].

While Noatak comprises the primary study area, a “modeling extent” consisting of a 20 km buffer around Noatak, clipped where it extends into the ocean (Fig. 1), is analyzed to avoid artificial boundary effects on estimates of landscape resistance and connectivity within Noatak [48, 58]. Koen et al. [48] recommend a buffer of approximately 20% of the study area width to remove the effects of node placement on current density. A 20 km buffer represents approximately 20% of the height of Noatak and 10% of the width and was chosen as a compromise between including a larger buffer and only including areas for which sport hunter usage data were available.

### Caribou telemetry data

Locations of 55 adult female caribou from the WACH were recorded using GPS telemetry data collected from 2010 to 2013. Caribou swimming across the Kobuk River at Onion Portage were restrained by hand and fitted with GPS telemetry collars programmed to record one location every eight hours [59]. Collars provided between one and four years of information (median = 2 years). Animal handling was approved by the State of Alaska’s Animal Care and Use Committee (ACUC) protocol #2012-031R.

Caribou data falling within the modeling extent were extracted for the autumn period (August 1^st^ to November 30^th^), corresponding with the primary migration and non-local hunting seasons. The resulting telemetry dataset contained 7006 total steps (pairs of locations). Steps with missing data (i.e., time intervals longer than eight hours) and those that had any part extending beyond the modeling extent were removed from the dataset. This resulted in 6596 steps over the 4-year period (94.2% of the original data).

### Environmental covariate data

Hunting activity data consisted of commercial aircraft transporter landing sites (remote, unmaintained strips used to get sport hunters in and out of Noatak) and sport hunter camps. Non-local hunters that utilized Noatak between 2010 and 2013 were asked to fill out a voluntary mail-back survey indicating the year and location of hunting camps they used [37]. Locations were converted to a spatial point dataset using ArcGIS (version 10.3, ESRI, Redlands, CA). Locations were only retained for hunting parties that spent at least 24 h within the modeling extent. Data on commercial transport aircraft landing events (locations and dates) were compiled from existing State of Alaska Big Game Commercial Services Board—Licensed Transporter Aircraft records and converted to a spatial point dataset in ArcGIS. Locations from the hunter camp and transporter landing datasets were clipped to the modeling extent and filtered to only include records occurring during the study period.

Predators, including human hunters, do not have to be physically present to affect the behavior and space use of prey species [60, 61]. This may lead to avoidance of areas easily accessible to hunters as a strategy to reduce predation risk [62]. To represent the general pattern of relative intensity of hunting activity to which caribou may be responding, a utilization distribution of hunting activity locations across the study period was created [63, 64]. The hunter camp and transporter landing datasets were combined into a single hunting activity layer for creation of the utilization distribution to account for spatial and temporal differences between the datasets and spatial autocorrelation. Hunter camp records indicated the year but not the specific date a camp was used. Because hunters were asked to report only the camp locations from their most recent trip, records were biased toward later years (number of camps in 2010 = 24, 2011 = 78, 2012 = 94, 2013 = 219). Transporter records were mixed, with some indicating the specific date of landing and others only the year. These locations were more evenly distributed over the study period (number of landings in 2010 = 140, 2011 = 190, 2012 = 216, 2013 = 152). Most hunters established camps near their drop-off locations (within 0.5 km) for the duration of their hunting trip, leading to a strong correlation between the transporter landing and hunter camp datasets. In addition, many hunters hunt on the day they are dropped off by the transporter. The utilization distribution on the combined dataset was created in R [65] at a 120 m spatial resolution using the adehabitatHR package [66] with the *ad hoc* approach of Kie [67] used to select the optimal bandwidth.

Other environmental variables included terrain ruggedness, river area, and land cover. While more rugged areas likely increase energetic costs for movement through climbing slopes [68] and greater movement tortuosity [69], previous studies have shown selection for more varied topography by caribou [59, 70], likely due to increased forage availability [71, 72]. Terrain ruggedness was calculated using the vector ruggedness measure, which measures vector dispersion on a digital elevation model (DEM) to take into account heterogeneity in both slope and aspect [73]. A 120 m DEM was derived from the National Elevation Dataset (NED; [74]). The 60 m NED elevation raster was resampled to a 120 m spatial resolution using cubic convolution. Terrain ruggedness was calculated on the 120 m resolution DEM in ArcGIS using the Vector Ruggedness Measure Tool [75] with a 3×3-pixel window. Williams and Gunn [76] investigated water crossings used by caribou in Canada and found river width appeared to influence crossing location, with caribou crossing more frequently in narrow areas. The percentage of a 120 m pixel consisting of river was used as a continuous raster representation of river width. River areas per pixel were calculated from the NHDArea polygons of the National Hydrography Dataset [77]. Land cover type was derived from a composite map of Alaska vegetation [78] and was grouped into eight classes: dwarf shrub, forest, tussock tundra, herbaceous, tall shrub, low shrub, lichen, and water. The water class was predominantly composed of lakes within the study area and only exhibited a 5.2% overlap with the river layer described above. The 30 m land cover data were resampled to a 120 m spatial resolution in ArcGIS using a majority filter. For maps and ranges of the input covariates see Additional file 1: Figure S1.

When resampling covariate data, the modifiable areal unit problem (MAUP) may be an issue, potentially affecting results based on the effect of changing spatial resolution rather than based on the ecological process of interest [79]. Unfortunately, there currently is a lack of reliable approaches to identify whether artifacts are introduced when working with real-world data [80]. In such situations, however, resampling may be necessary when combining disparate datasets with varying native resolutions to provide a uniform scale for resistance surface creation. Further constraints on resolution may be presented by analytical issues such as maintaining computational feasibility for connectivity models. Fortunately, some connectivity approaches appear to be robust to altering resistance surface resolution (e.g., [45]).

### Statistical analysis

Caribou movement was analyzed following the step selection function randomized shortest path (SSF-RSP) approach of Panzacchi et al. [53]. The SSF-RSP approach first uses step selection function (SSF) models to estimate resistance to movement from environmental variables. Following Panzacchi et al. [53], we used conditional logistic regression to compare the environmental covariates for each observed caribou step against ten randomly generated “available” steps. How availability data are defined in such studies can strongly influence findings [81]. Lengths of random steps were chosen using a uniform distribution with a maximum corresponding to the 99^th^ percentile of the observed step length distribution to avoid inclusion of too many points close to the starting location [53, 82]. Continuous variables (e.g., terrain ruggedness, hunting activity) were recorded as the maximum value along a step [53]. Since the goal was to estimate resistance to movement, the maximum value was used to represent the limiting value of a given variable along a movement step. Categorical land cover classes were also represented in a continuous manner as the proportion of each land cover class along a step. Step lengths were included in the conditional logistic regression model to reduce bias in model coefficients [83] and were adjusted for elevation to better reflect the total distance travelled in a step [53]. Covariates were standardized by dividing by twice their standard deviation [84]. Analysis of variance inflation factors showed collinearity was not an issue (all VIF values < 3; [85, 86]), so a set of candidate SSF models was built using a factorial combination of environmental covariates (Table 1). All models contained a spline of the elevation-adjusted step length, included using the pspline function in the survival package [87, 88] with two degrees of freedom, following Panzacchi et al. [53].

**Table 1:** Candidate models for caribou resource selection in Noatak National Preserve, Alaska

Model number	Model
0	Null
1	Rugged
2	Hunting
3	River
4	LandCover
5	Rugged + Hunting
6	Rugged + River
7	Rugged + LandCover
8	Hunting + River
9	Hunting + LandCover
10	River + LandCover
11	Rugged + Hunting + LandCover
12	Rugged + Hunting + River
13	Rugged + River + LandCover
14	Hunting + River + LandCover
15	Rugged + Hunting + River + LandCover

Covariates considered included terrain ruggedness (Rugged), sport hunting activity (Hunting), river area (River) and land cover type (LandCover). LandCover consisted of seven parameters, representing the proportion of each land cover type. In addition, each candidate model included a spline of the distance to previous used location to help reduce bias in step selection function estimation

Conditional logistic regression (CLR) models were run using the survival package and model selection was performed using Akaike’s Information Criterion corrected for small sample size (AICc; [89]). Robust standard errors controlling for multiple observations per individual were calculated following the approach of Forester et al. [83]. In brief, this consisted of fitting an intercept-only mixed-effects model to the deviance residuals of the top SSF model, with a random intercept included for individual caribou. The lag of correlation was identified using an autocorrelation function on the mixed-effects model results and was used to assign the original data into independent clusters. The data were subset into two independent groups using the clusters and a CLR model was fit on each subset. The resulting covariance matrices from each subset were then averaged to provide adjusted standard errors. Predictive performance of the final model was evaluated using k-fold cross validation [90, 91] with ten folds.

The final model from the SSF analysis was used to predict movement friction (1/relative suitability) across the study area. This friction map was then input into a randomized shortest path (RSP) model to predict the presence of corridors and barriers across the landscape under various movement strategies [53]. The RSP model estimated the expected number of passages of animals moving between two areas under different movement strategies [53]. Movement strategies were controlled by a parameter, *θ*, that influenced how strongly the modeled animals tended between a random walk movement model (habitat exploration) and a least cost path movement model (habitat exploitation; for details see [53]). Multiple RSP models were run, testing different values for *θ* to reflect varying tradeoffs between exploration and exploitation. Seven *θ* values were modelled: 0, 1e-6, 1e-5, 1e-4, 1e-3, 1e-2, 1e-1. The endpoints represented random walk (exploratory) and least cost path (exploitative) movement, respectively, while the intermediate values represented a mix between the two approaches. For each *θ* value 100 random pairs of points were generated with one point in the northern half of the 20 km buffer around Noatak and the other point in the southern half of the buffer. An RSP model was run for each pair and the resulting maps were summed to yield an overall estimate of number of visits per pixel across the 100 pairs. R code to run the SSF and RSP models was adapted from Panzacchi et al. [53]. RSP analyses were run for the full modeling extent and resulting maps were clipped to the Noatak boundary study area to remove edge effects from the modeling process.

The best fitting RSP *θ* value was selected as the one that minimized the mean squared error (MSE) between the RSP model and observed caribou corridors represented by a Brownian bridge movement model (BBMM; [53]). A BBMM was created using the BBMM package in R [92], which follows the general approach of Horne et al. [93] and Sawyer et al. [94]. Location error in the BBMM model was parameterized as 33 m [95]. RSP outputs and BBMM maps were standardized to sum to one before MSE calculation [53]. The summed map from the RSP models with the best-fitting *θ* value provided a population-level representation of relative suitability for migration across the study area.

## Results

Model selection indicated two models had ΔAICc values of less than 2 (Table 2). As Burnham and Anderson [89] pointed out,

**Table 2:** Model selection results for caribou resource selection in Noatak National Preserve, Alaska

Model	K	ΔAICc	Akaike weight	Log-likelihood
13	16	0.00	0.62	-11721.49
15	17	0.94	0.38	-11720.96
7	15	16.82	0.00	-11730.90
11	16	17.31	0.00	-11730.15
10	15	85.96	0.00	-11765.47
14	16	87.94	0.00	-11765.46
4	14	94.00	0.00	-11770.49
9	15	95.94	0.00	-11770.46
6	9	135.32	0.00	-11796.16
12	10	136.76	0.00	-11795.88
1	8	158.33	0.00	-11808.66
5	9	159.41	0.00	-11808.20
3	8	245.84	0.00	-11852.42
8	9	247.49	0.00	-11852.24
2	8	259.20	0.00	-11859.09
0	0	123552.79	0.00	-73513.89

Candidate models were compared using Akaike’s Information Criterion adjusted for small sample size (AICc). The number of parameters retained for each model (K), difference in AICc values between models (ΔAICc), corresponding Akaike weights, and maximized log-likelihoods of each model are reported here. Model numbers correspond to Table 1

“Models having Δ_*i*_ within about 0–2 units of the best model should be examined to see whether they differ from the best model by 1 parameter *and* have essentially the same values of the maximized log-likelihood as the best model. In this case, the larger model is not really supported or competitive, but rather is ‘close’ only because it adds 1 parameter and therefore will be within 2 Δ_*i*_ units, even though the fit, as measured by the log-likelihood value, is not improved” (p.131).

In such cases, the additional parameter in the larger of the two models is referred to as an ‘uninformative parameter’ and should not be considered to be supported by model selection [96]. This was the case in our study. The top two models, Model 13 and Model 15, differed by a single parameter – hunting activity (see Table 1 for model details). The maximized log-likelihood values were very similar, differing by only 0.53 (Table 2). The similar log-likelihoods of the top two models and difference of a single parameter indicated that hunting activity provided an uninformative parameter in Model 15, thus leading us to follow the recommendation of Arnold [96] and discard this model. This was affirmed by investigation of the confidence intervals for hunting activity. Both the 85% (which are more compatible with use of AICc for model selection [96]) and 95% confidence intervals for hunting activity overlapped zero. The final SSF model used in our analyses, Model 13, thus included parameters for terrain ruggedness, river area, land cover type, and elevation-adjusted step length, but not hunting activity.

Step selection analysis revealed areas with more rugged terrain and greater river area had a lower likelihood of use by migrating caribou (Table 3). Areas with a higher proportion of dwarf shrubs and tussock tundra were more likely to be used by migrating caribou, while those with a higher proportion of forest, tall shrubs, and water were more likely to be avoided. Herbaceous vegetation and areas dominated by lichens were used proportionally to their availability. Cross-validation results indicated high predictive performance for the final model (mean Spearman’s correlation across folds = 0.985).

**Table 3:** Conditional logistic regression coefficients for the final model of step selection by caribou in Noatak National Preserve, Alaska

Covariate	Coefficient	Standard Error
**Terrain ruggedness**	**-0.62**	**0.12**
**River area**	**-0.22**	**0.08**
**Dwarf shrub proportion**	**0.19**	**0.08**
**Forest proportion**	**-0.35**	**0.09**
Herbaceous proportion	0.06	0.05
Lichen proportion	-0.10	0.08
**Tall shrub proportion**	**-0.22**	**0.08**
**Tussock tundra proportion**	**0.19**	**0.09**
**Water proportion**	**-0.11**	**0.05**
**Step length 1**	**-2.43**	**0.13**
**Step length 2**	**-4.66**	**0.23**
**Step length 3**	**-5.94**	**0.24**
**Step length 4**	**-5.48**	**0.30**
**Step length 5**	**-4.15**	**0.54**
**Step length 6**	**-2.70**	**0.86**
Step length 7	-1.23	1.19

The Step length 1–7 covariates report the coefficient values from the elevation-adjusted step length spline. Standard errors reflect Forester et al. [83]’s adjustment for serial autocorrelation. Values in bold indicate that the coefficient’s 95% confidence interval does not overlap zero. Overlap patterns were identical for 85% confidence intervals (cf. Arnold [96] for use of wider confidence intervals with information theoretic model selection)

**Fig. 2:**
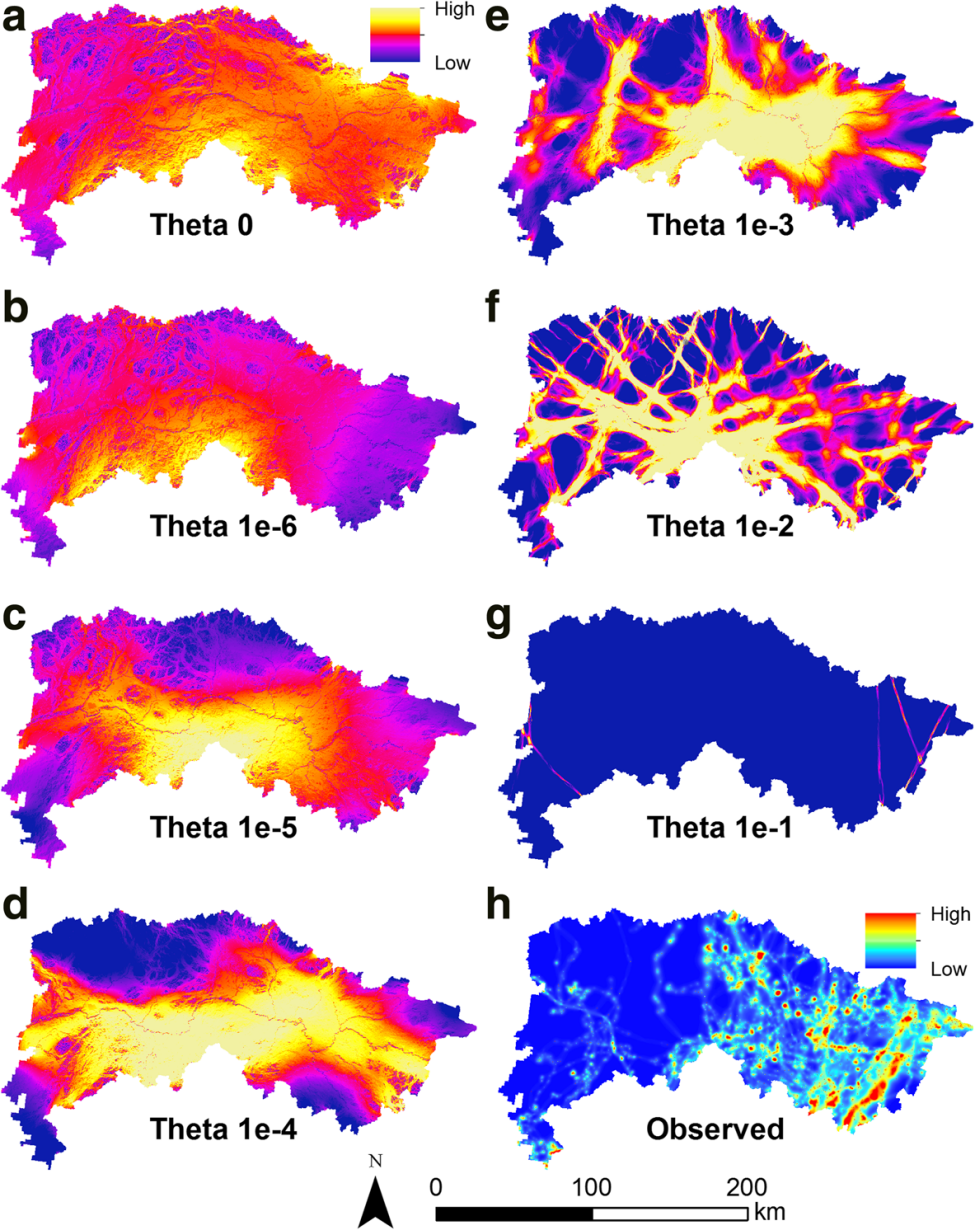
Predicted and observed corridors for autumn caribou movement through Noatak National Preserve, 2010–2013. Predicted corridor maps (**a–g**) are the sum of 100 randomized shortest path model runs for a given *θ* value. Values of *θ* represent a range of movement strategies with *θ* = 0 reflecting random-walk style exploratory movement (**a**) and *θ* = 0.1 reflecting least cost path exploitative movement (**g**). Intermediate *θ* values depict a mixture between these two strategies. Observed movement corridors (**h**) are represented using a Brownian bridge movement model built on GPS locations of 55 adult female caribou

Summed RSP maps for the seven *θ* values covered a wide range of spatial patterns (Fig. 2a–g). Predicted distributions displayed by the RSP maps followed expected patterns under the movement strategies represented by the various *θ* values. Maps of smaller *θ* values featured broad areas of connectivity, reflective of spatial use under a random walk, while those for larger values of *θ* tended to strongly concentrate corridors into relatively straight lines, reflecting least cost movement. Intermediate *θ* values showed a gradient between these two patterns. Calculation of the mean squared error (MSE) between RSP maps and observed caribou corridors (Fig. 2h) identified a *θ* value of 0 as the best-fitting model (Table 4). This indicated that caribou passing through Noatak in autumn exhibit primarily exploratory movement, following predictions of a random walk model. Under this model, predicted caribou use was broad across the study area, but concentrated most strongly in the central and eastern portions of Noatak and was relatively lower in the western area of the preserve (Fig. 2a; Additional file 1: Figure S2).

**Table 4:** Mean squared error values reflecting fit between predicted and observed movement corridors

*θ* value	Mean squared error
0	5.08 × 10^-13^
1 × 10^-6^	5.28 × 10^-13^
1 × 10^-5^	5.10 × 10^-13^
1 × 10^-4^	5.22 × 10^-13^
1 × 10^-3^	5.21 × 10^-13^
1 × 10^-2^	5.65 × 10^-13^
1 × 10^-1^	3.11 × 10^-12^

Predicted movement corridors were generated using randomized shortest paths (RSPs) for each *θ* value. A *θ* value of 0 reflects exploratory movement, similar to a random walk model, while a value of 0.1 reflects exploitative movement, similar to least cost path models. Observed movement corridors were based on a Brownian bridge movement model of caribou telemetry data. Smaller mean squared error values reflect better fit between models

## Discussion

Our analysis of caribou movement in Noatak National Preserve shows that caribou respond to environmental features such as terrain ruggedness and land cover type, but not to sport hunting activity at the scale considered. The negative effect of terrain ruggedness on caribou movement aligns with patterns seen for the WACH at the scale of the full autumn migratory path (Fullman et al., in revision), though in winter, when they are non-migratory, caribou may select for more rugged terrain [27, 70]. Similarly, our finding that caribou avoid migratory pathways with greater river area aligns with caribou crossing more frequently in narrow portions of rivers in Canada [76] and with increased landscape resistance to autumn migratory movement from major rivers for the WACH (Fullman et al., in revision). Patterns of vegetation influence on step selection also coincide with other reports of avoidance of dense vegetation by caribou ([70, 97], Fullman et al., in revision). Avoidance of dense vegetation may be to facilitate travel and/or to reduce predation risk.

We did not detect an effect of sport hunting activity on caribou resource selection, supporting our null hypothesis. This indicates that sport hunting does not inhibit the ability of caribou to migrate through Noatak. Local hunters have harvested caribou at key river crossing locations for 10,000 years in northwest Alaska [98]. That these locations continue to be used by caribou and local hunters to this day [24] may support our findings. Further, studies elsewhere have also found environmental factors have a greater impact on animal space use than hunting (e.g., [61, 62]). Our finding of a lack of effect of sport hunting activity on the likelihood of caribou migrating through Noatak does stand in apparent contrast to concerns voiced by local hunters regarding the negative effects of sport hunters and commercial air transporters (e.g., [39, 42, 43]). These differences may relate to issues of spatial and temporal scale. The caribou GPS locations in this study were recorded every eight hours. It is possible that caribou response to human hunting activity is short-lived, such that it is not detectable at an eight-hour interval. Experimental studies of woodland caribou response to simulated seismic exploration found that caribou respond to noise disturbance by increasing movement rates for between 15 min and at least two hours [99–101]. They also reported that linear displacement distances from the point of disturbance were not significantly different from control animals [100]. Such temporary responses could still have an influence for a local hunter waiting for caribou to approach, but may not affect the ability of caribou to pass through Noatak and thus not be reflected in our analysis. In addition, the lack of observed influence of sport hunting activity on caribou movement through Noatak may reflect differences in scale between the caribou telemetry dataset, which was recorded sub-daily, and the sport hunter dataset, which was aggregated across years. An aggregated representation of the general likelihood of encountering people may not reflect the fine scales at which caribou respond to and avoid hunting activity. Studies utilizing finer spatial and temporal resolution data may be needed to detect the effects described by local hunters. Furthermore, no data were available on locations of local hunting activity, so it is unclear what effect this may have had on caribou movements in areas not used by sport hunters or whether there may be additive effects. Finally, changes in the migratory routes of WACH caribou described by local residents [42] may be related to an industrial road that bisects the herd’s migration route to the east of the study area [102]. In summary, our findings clearly indicate that sport hunting activity does not prevent caribou from migrating through Noatak, but do not address possible impacts of sport hunting disrupting individual subsistence hunting attempts at fine spatial and temporal scales.

In addition to movement responses to environmental covariates, the SSF-RSP approach also provides information on the movement strategy used by caribou in crossing through Noatak. Comparison of predicted and observed movement corridors indicates that caribou moving through Noatak are employing random walk movement behavior. This is in contrast to reindeer in Norway, which appear to follow a mixed movement strategy intermediate between random walk and least cost path movement patterns [53]. One possible explanation for the adoption of exploratory movement behavior we observed is recent patterns of mild, long autumn seasons and relatively warm winters during the study period [103]. The body mass of female caribou has a strong influence on the likelihood of successfully giving birth, timing of birthing, birth mass of calves, and offspring survival [101, 104, 105]. In elk, females that consistently select the highest quality forage available in the autumn enter winter in better condition than other individuals [106]. Mild autumn and winter conditions may allow caribou in northwestern Alaska to adopt a similar strategy, employing predominantly exploratory movement behavior to capitalize on available resources and improve body condition prior to winter. The Arctic has been warming recently [107, 108], with temperature increases most pronounced in the winter [109]. If these trends continue, mild conditions during autumn migration may become more common, reinforcing the effectiveness of an exploratory movement strategy for caribou seeking to improve body condition prior to winter.

Although not tested in this study, it is possible that our observation of exploratory movement behavior for female caribou passing through Noatak is season-specific. Female caribou migrate to the calving grounds quickly in the spring, while males lag behind to forage on new growth [59, 110, 111]. Rapid migration by pregnant females in the spring may encourage exploitative movement behavior with straighter movement paths, such as are expected with a least cost path approach. Indeed, Bergman et al. [112] observed straightened movement paths during migration to calving grounds in Canadian caribou. The mixed movement strategies reported for reindeer in Norway were observed during spring migration [53], which might contribute to the different pattern observed in our study. McClure et al. [54] found that different modeling strategies (circuit theory versus least cost paths) may be more applicable for certain types of movement than others (e.g., migration versus dispersal) and thus that the modeling approach should be selected based on the movement ecology of the focal species of interest. We suggest that this may be true even within a movement process based on sex- or season-specific influences on motivators and constraints of movement. Comparative studies are needed that explore this possibility more thoroughly.

## Conclusions

Understanding animal movement behavior is fundamental to protecting critical areas of connectivity and to informing management decisions. We use step selection functions and randomized shortest paths to investigate how female caribou moving through Noatak respond to non-local hunting activity, terrain ruggedness, rivers, and land cover. Our results indicate that non-local hunting activity does not appear to affect the ability of caribou to pass through Noatak, though this does not preclude the possibility of fine-scale or temporary effects altering availability of caribou for local hunters. In addition, we found that caribou moving through Noatak employ random walk movement behavior. Such exploratory movements may reflect a behavioral response taking advantage of recent mild autumn and early winter conditions to increase body condition prior to winter, improving the likelihood of successful reproduction. Our findings have direct and immediate management implications as federal public lands in Alaska, including Noatak, have been closed to sport hunters seeking to harvest caribou due in large part to local perception that these users were negatively impacting caribou migration. The closure follows the implementation of a delayed entry area for non-local hunters in Noatak that failed to resolve user conflicts in this conservation unit.

## Additional file

Additional file 1: Figure S1. Input covariate data for analyzing step selection by caribou in Noatak National Preserve, Alaska. **Figure S2.** Best-fitting connectivity map for caribou in Noatak National Preserve, Alaska. (PDF 540 kb)
