# Supplementary material for: Effects of environmental features and sport hunting on caribou migration in northwestern Alaska
Source: Mov Ecol. 2017 Mar 1;5:4. doi: 10.1186/s40462-017-0095-z (PMC5331706; doi:10.1186/s40462-017-0095-z)
Supplement: Additional file 1: Figure S1. — Input covariate data for analyzing step selection by caribou in Noatak National Preserve, Alaska. Figure S2. Best-fitting connectivity map for caribou in Noatak National Preserve, Alaska. (PDF 540 kb) [file 40462_2017_95_MOESM1_ESM.pdf]

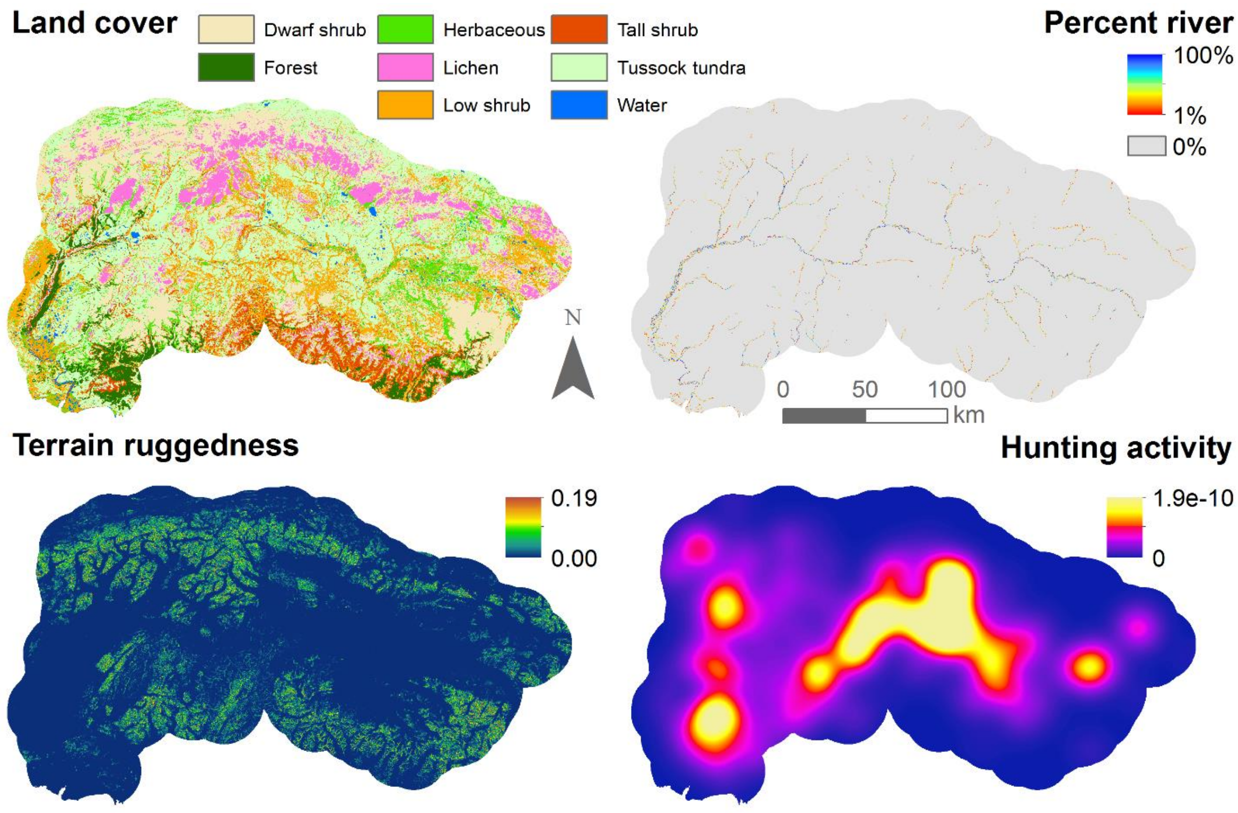

Fig S1. **Input covariate data for analyzing step selection by caribou in Noatak National Preserve, Alaska.** Caribou resource selection was evaluated for four environmental covariates: land cover type, the percentage of river per pixel (river area), terrain ruggedness, and hunting activity. Terrain ruggedness reflects the vector ruggedness measure of Sappington et al. (2007), which produces a unitless measure ranging from 0 to 1 with higher values indicate more rugged terrain (greater heterogeneity of slope and aspect). Hunting activity represents a utilization distribution depicting the relative frequency of use by aircraft transporters and hunting camps across the study area.

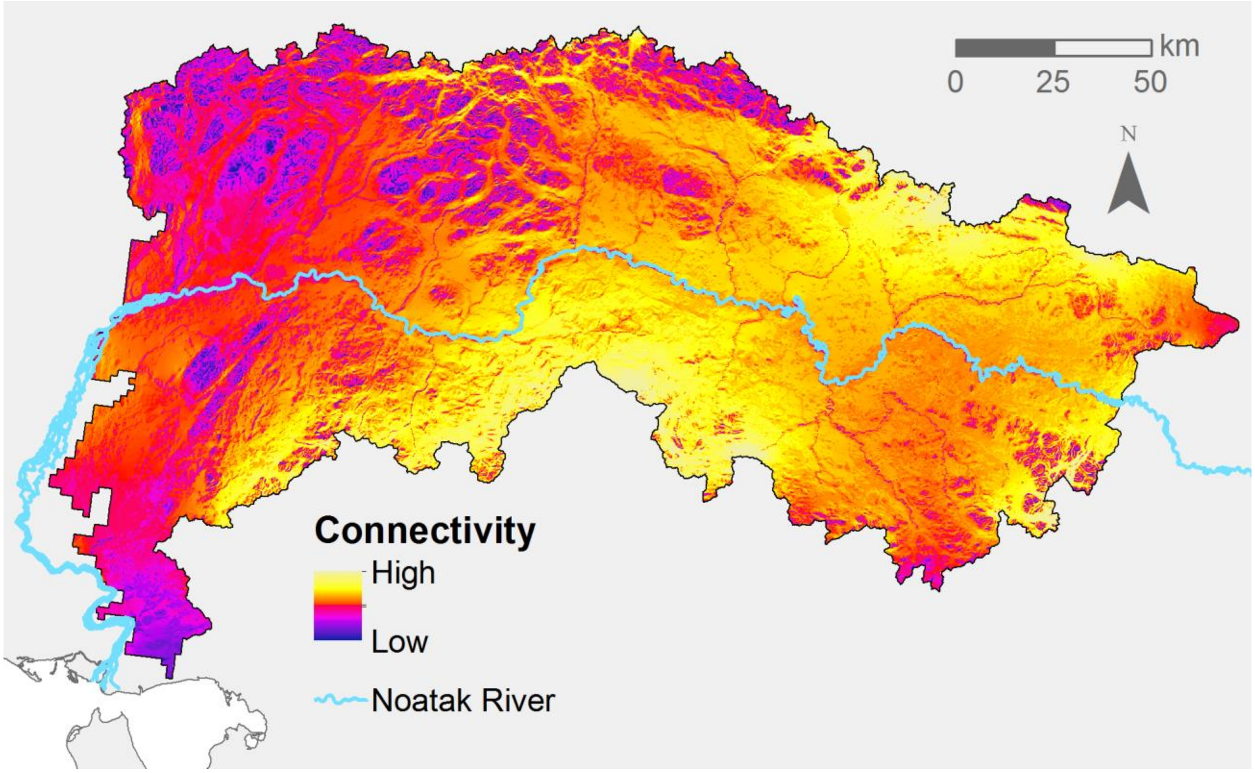

Fig S2. **Best-fitting connectivity map for caribou in Noatak National Preserve, Alaska.** Connectivity maps were calculated using the randomized shortest path approach of Panzacchi et al. (2016). A θ value of zero produced the minimum mean squared error when compared against observed caribou corridors (see Fig. 2 and Table 4). This corresponds to exploratory movement following a random-walk. The map depicts the same data as in Fig. 2a but with a histogram stretch specific to the displayed data. The location of the Noatak River is indicated for reference.

References

Panzacchi M, Van Moorter B, Strand O, Saerens M, Kivimäki I, St. Clair CC, Herfindal I, Boitani L. Predicting the continuum between corridors and barriers to animal movements using Step Selection Functions and Randomized Shortest Paths. J Anim Ecol. 2016;85:32–42.

Sappington JM, Longshore KM, Thompson DB. Quantifying landscape ruggedness for animal habitat analysis: a case study using bighorn sheep in the Mojave Desert. J Wildl Manage. 2007;71:1419-26.
